# Fertility Challenges: The Complexities of Retrograde Ejaculation and Cornual Block in Reproductive Health

**DOI:** 10.7759/cureus.55523

**Published:** 2024-03-04

**Authors:** Maitrey N Kasbe, Akash More, Gauri Gajabe, Ritesh Jadhav, Praful Bachate, Saurabh Mehakar, Princee Tyagi

**Affiliations:** 1 Clinical Embryology, Datta Meghe Institute of Higher Education & Research, Wardha, IND; 2 Anatomy, Datta Meghe Institute of Higher Education & Research, Wardha, IND; 3 Interventional Radiology, Datta Meghe Institute of Higher Education & Research, Acharya Vinoba Bhave Rural Hospital, Wardha, IND

**Keywords:** tubal disorder, tesa, cornual block, retrograde ejaculation, infertility

## Abstract

Infertility, a complicated reproductive health issue that affects both men and women, can have a variety of causes, from anatomical abnormalities to hormone imbalances. This research addresses a couple who have been struggling with infertility for the past four years: a 31-year-old woman with bilateral tubal blockage and her 34-year-old spouse who suffered from primary infertility due to retrograde ejaculation (RE) for the same period. Analyzing the male’s semen sample, it was discovered that there were dead sperm and urine, indicating RE. A hysterosalpingography indicated bilateral tubal obstruction in the female partner. Pelvic factors were examined via laparoscopy, which played a crucial role in addressing further issues. The procedure of treatment included testicular sperm aspiration for sperm extraction and intracytoplasmic sperm injection. Hormonal support was involved in the follow-up, and on the 14th day, the β-hCG test came back positive. The intricate procedures of RE and cornual block are discussed, with a focus on how they affect reproductive health.

## Introduction

Infertility is a condition of the male or female reproductive system defined by the failure to achieve a pregnancy after 12 months or more of regular, unprotected sexual intercourse. It results from various factors that may affect both men and women, encompassing issues related to reproductive organs, hormonal imbalances, genetic factors, and lifestyle choices. Causes of infertility can range from ovulatory disorders and tubal blockages in women to sperm abnormalities and erectile dysfunction in men [1]. The process of seminal fluid being forced into the bladder from the posterior urethra is known as retrograde ejaculation (RE). The inner longitudinal and outer circular smooth muscle layers that make up the posterior urethra are a direct extension of the bladder’s inner and outer longitudinal muscles, respectively. RE can occur when the regular ejaculatory system malfunctions [2]. Not every individual with RE will be able to recover normal antegrade ejaculation, despite the possibility of curing it surgically or with medication. However, the need for the absence of urine in the bladder at ejaculation is repeatedly stressed [3]. RE is unusual in that it is almost entirely organic in origin, unlike many ejaculatory illnesses that might have both psychological and organic roots. Although it is a prevalent kind of ejaculatory dysfunction, only 0.3-2% of cases of infertility are caused by it.

Upon ejaculation, sperm are rapidly taken along the vas deferens and into the urethra via the ejaculatory ducts. From there, the semen progresses in an antegrade fashion, partly maintained by coaptation of the bladder neck and rhythmic contraction of the periurethral muscles coordinated by a centrally mediated reflex. The sympathetic nervous system brings about the seminal emission and closure of the bladder neck, which begins with the limber sympathetic ganglia and ends with the hypogastric nerve. The parasympathetic nervous system initiates prostatic and seminal vesicle secretion and contraction of the bulbocavernosus, ischiocavernosus, and pelvic floor via the pelvic nerve [4]. The cornua, often known as uterine horns, is a term for the uterine attachment of the fallopian tubes. Infection can obstruct the cornua, resulting in a cornual block. It could be the primary or secondary cause of infertility. A cornual obstruction of the fallopian tubes results in a cornual spasm or contraction. Approximately 75% of the fallopian tubes are covered with it. Cornual block causes the fallopian tubes to become blocked, which restricts sperm entry and prevents the ovum from being fertilized. Tubal disorders affect about 35% of women who are infertile [5]. The fallopian tube is 7-9 cm, with an elongated shape like a trumpet that spreads from the cornua of the uterine cavity to the ovary. Its fimbriated end, which opens to the peritoneal cavity, curves over the ovary, pulling the ovulated egg into the fallopian tube, where fertilization occurs [6].

## Case presentation

A 31-year-old female with the issue of blockage in the fallopian tube came to the infertility clinic with her 34-year-old husband, who was experiencing infertility issues of RE. The couple had been trying to conceive for four years. This was the first time that the couple sought infertility treatment. There was a past medical history of smoking, alcohol consumption, etc., in the husband. The female had no past medical history of endometriosis or pelvic surgery, and the gravida stage was 0. Her menses were irregular, and she had no history of dyspareunia. No relevant findings were found during the physical examination, where the male’s BMI was 23 kg/m^2^, and the female’s BMI was 22 kg/m^2^.

Investigations

In the semen analysis report, the presence of urine and dead sperm was observed in the husband’s ejaculation. This led to the inference that the husband had the issue of RE. The female partner underwent a transvaginal ultrasound to explore and identify any structural abnormalities. The anti-Müllerian hormone level of the female averaged around 1.3 ng/ml, the female follicle-stimulating hormone (FSH) level was <5.6 mIU/ml, and the luteinizing hormone level was 2.50 mIU/ml. To diagnose the tuber blockage in the female, hysterosalpingography (HSG) was performed. The investigation revealed that the patient was suffering from bilateral tubal blockage.

Diagnosis

**Figure 1:**
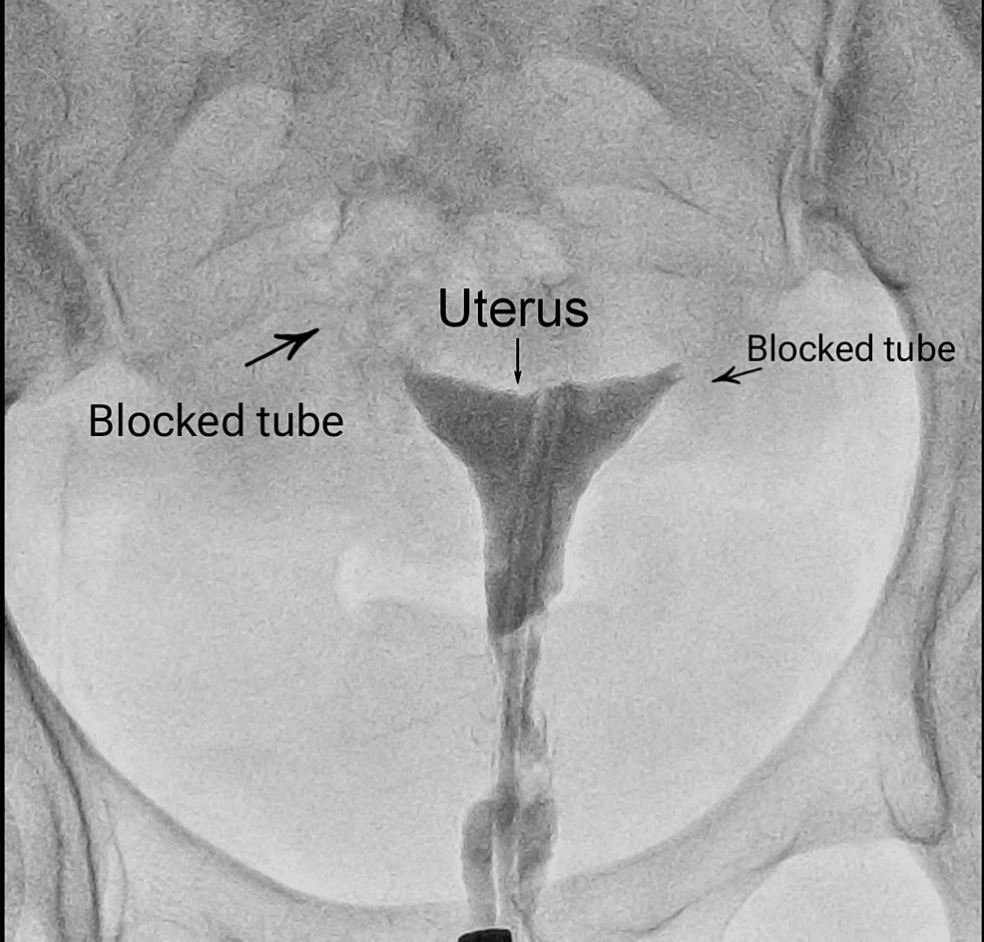
HSG of the female partner showing bilateral tubal blockage HSG, hysterosalpingography

The couple faced primary infertility, with the potential cause identified in the male as RE. HSG was employed to examine the fallopian tube from the inside to aid in diagnosing blockage. The investigation revealed bilateral tubal blockage in the female, as illustrated in Figure 1.

During the initial semen analysis, the sperm count was 1.3 million per milliliter, with a motility of 32%, and normal morphology was 22%, as indicated in Table 1. Subsequent second and third semen analyses were conducted at a two-week interval, revealing no significant changes, as shown in Table 1.

**Table 1:** Semen analysis of the male partner

Parameter	Sample 1	Sample 2	Sample 3	Normal range
Volume (ml)	0.8 ml	1.0 ml	1.3 ml	1.5 ml
pH	7.3	7.1	7.2	7.2-8
Sperm count (million/ml)	1.3 million/ml	1 million/ml	1.1 million/ml	15 million/ml
Normal morphology	6%	15%	7%	Apr-14
Sperm motility (%)	32%	28%	25%	40%

Treatment 

Through HSG, the tubal blockage was assessed. Both fallopian tubes were found to be blocked. The FSH level was low, i.e., 2.8 IU/1, indicating that tubal factors were causing infertility. The patient underwent laparoscopic surgery to address the tubal blockage, resulting in the clearance of 80% of the blockage, with 20% remaining. Subsequently, ovum pickup or oocyte retrieval was scheduled to collect mature eggs, followed by in-lab fertilization with sperm. Four oocytes were retrieved, comprising two at the meiosis II stage, one at the meiosis I stage, and one at the germinal vesicle stage.

Following the initial semen analysis and with no improvement in sperm count observed after two weeks, the patient was counseled for the testicular sperm aspiration (TESA) procedure. The patient’s consent was obtained for TESA, a technique employed when sperm cannot be obtained through ejaculation. TESA involves extracting sperm directly from the testis using a needle, typically performed under local anesthesia.

During the TESA procedure, an 18-G needle inserted in a 10-ml syringe loaded into a fine needle aspiration gun, along with 1 ml of sperm buffer, was used. Tissue samples were aspirated, and approximately 1 ml of buffer was utilized to optimize the retrieval process. The collected tissues were transported to the lab. Under microscopic examination, viable sperm were identified and isolated from the tissue sample, while debris was discarded. This purification process ensured that the retrieved sperm samples were suitable for assisted reproductive technology. The extracted sperm was then utilized for the intracytoplasmic sperm injection procedure.

Follow-up

After the embryo transfer, the female patient underwent a supplement regimen of 2 mg of estrogen consumed three times a day and 2 mg of progesterone taken twice a day, with the goal of supporting early pregnancy and creating an environment that was suitable for implantation. On the 14th day following the transfer, the β-hCG test was performed; the hormone level was 1023 mIU/ml, showing a favorable result. This important turning point indicated the successful implantation of the embryo and the start of the pregnancy, requiring close observation and extra care to maximize the health of both the mother and the fetus as the pregnancy developed.

## Discussion

This discussion examines two specific aspects of infertility: RE and cornual block, examining their causes, mechanisms, and impact on reproductive health [7]. RE, the process where seminal fluid is forced into the bladder instead of being expelled through the penis, is a unique condition that disrupts the regular ejaculatory system. The intricate coordination of smooth and striated muscles in the posterior urethra plays a crucial role in the ejaculation process [8]. The malfunctions of this system can result from congenital or acquired anomalies of the bladder, neck, and urethra, as well as interference with the neurologic control of ejaculation, especially in conditions like diabetes. Various factors contribute to RE, including the use of certain medications like adrenergic-stopping drugs [9]. Notably, not all cases can be successfully treated to restore normal antegrade ejaculation. Treatment options range from surgical interventions to medication and even pre-ejaculatory bladder washouts with buffers or saline. The stress and emotional toll on individuals and their partners experiencing RE, particularly in the context of fertility issues, are highlighted. Despite being a prevalent ejaculatory dysfunction, RE accounts for only 0.3-2% of infertility cases. Its predominantly organic origin sets it apart from many other ejaculatory disorders, which often have both psychological and organic roots [10]. Understanding the standard mechanism of ejaculation is crucial to comprehending the deviations that lead to conditions like RE. The coordination between the sympathetic and parasympathetic nervous systems and the involvement of various anatomical structures, such as the prostate, seminal vesicles, and bladder neck, is essential for successful ejaculation [11]. Disruptions in this intricate coordination can result in infertility. Shifting focus to female infertility, cornual block, a condition where infection obstructs the uterine horns (cornua), is explored. This blockage, whether primary or secondary, can significantly impact fertility, restricting sperm entry and preventing fertilization. Tubal disorders, affecting approximately 70% of infertile women, often involve the fallopian tubes, which are crucial for the transport of eggs and fertilization. A detailed look at the anatomy of the fallopian tube, a 7-9 cm elongated structure resembling a trumpet, emphasizes its pivotal role in reproduction. From the cornual attachment to the uterine cavity to the fimbriated end that curves over the ovary, the fallopian tube facilitates the journey of the ovulated egg, where fertilization takes place [12].

## Conclusions

This study highlights the complexities of RE and cornual blockage and provides valuable insights into the multifaceted nature of infertility. Through the use of modern diagnostics and cooperative strategies, we provide new hope to couples who are trying to overcome these challenges. An enhanced understanding of the causes and mechanisms of these conditions is essential for developing effective treatments and interventions to address infertility in both men and women. This report shows the need to continuously seek creative methods and provide care as they prepare to become parents.
